# Supplementary figures and images for: Mycobacterium tuberculosis resides in lysosome-poor monocyte-derived lung cells during chronic infection
Source: PLoS Pathog. 2024 May 3;20(5):e1012205. doi: 10.1371/journal.ppat.1012205 (PMC11095722; doi:10.1371/journal.ppat.1012205)

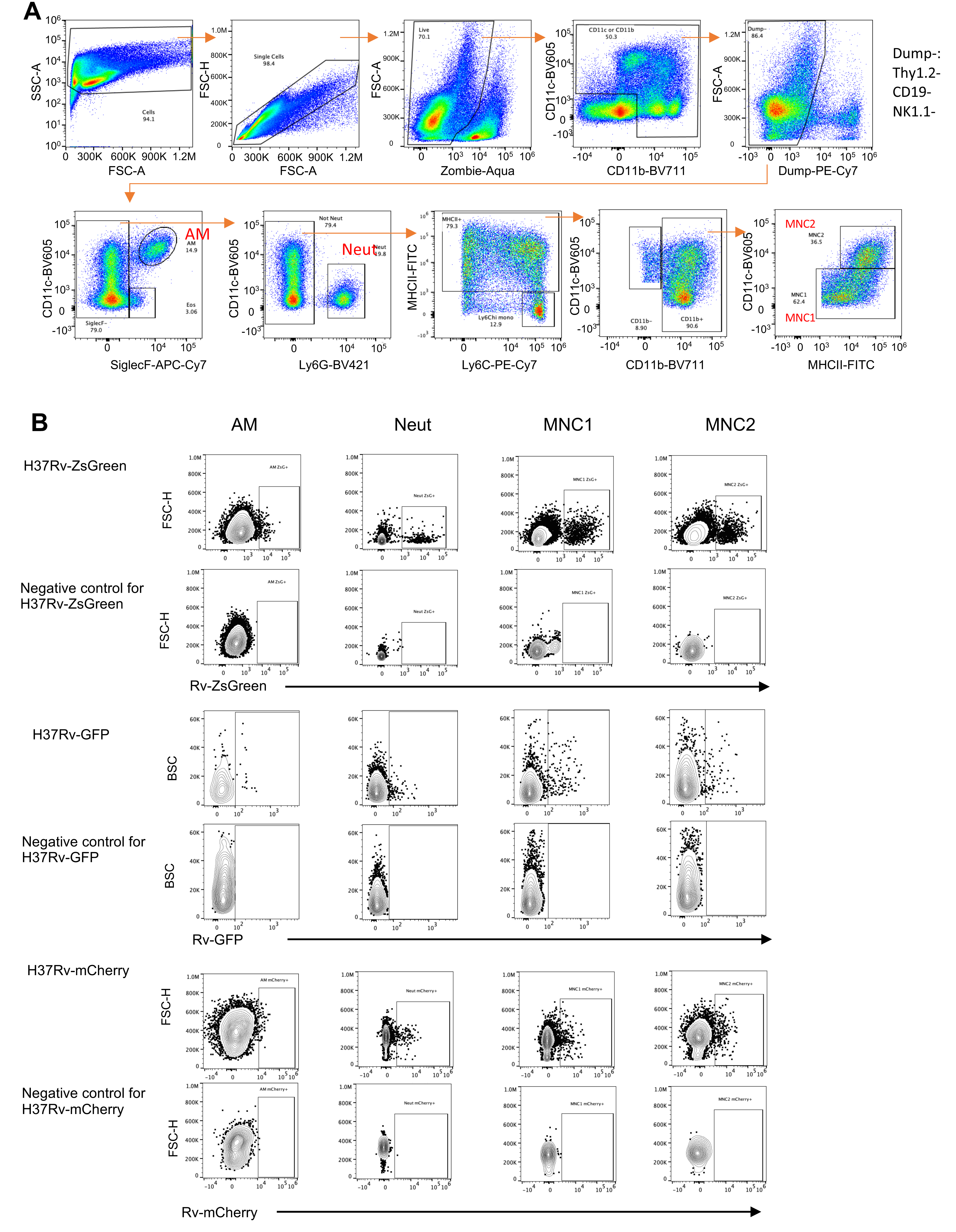

Supplement: S1 Fig — (A) The representative flow panel to detect AM, neutrophil (Neut), MNC1, and MNC2 populations in lungs from mice infected with H37Rv-mCherry (28 dpi). After gating out B, T, and NK cells, AM are CD11bloCD11chiSiglecFhi, MNC1 are SiglecF-CD11b+CD11cloMHCII+, MNC2 are SiglecF-CD11b+CD11chiMHCIIhi, and neutrophils (Neut) are SiglecF-Ly6GhiCD11bhi. (B) Illustrative plots of infected lung cells in each subset from mice infected with H37Rv-ZsGreen, H37Rv-GFP, or H37Rv-mCherry (28 dpi). (TIF) [file ppat.1012205.s001.tif]

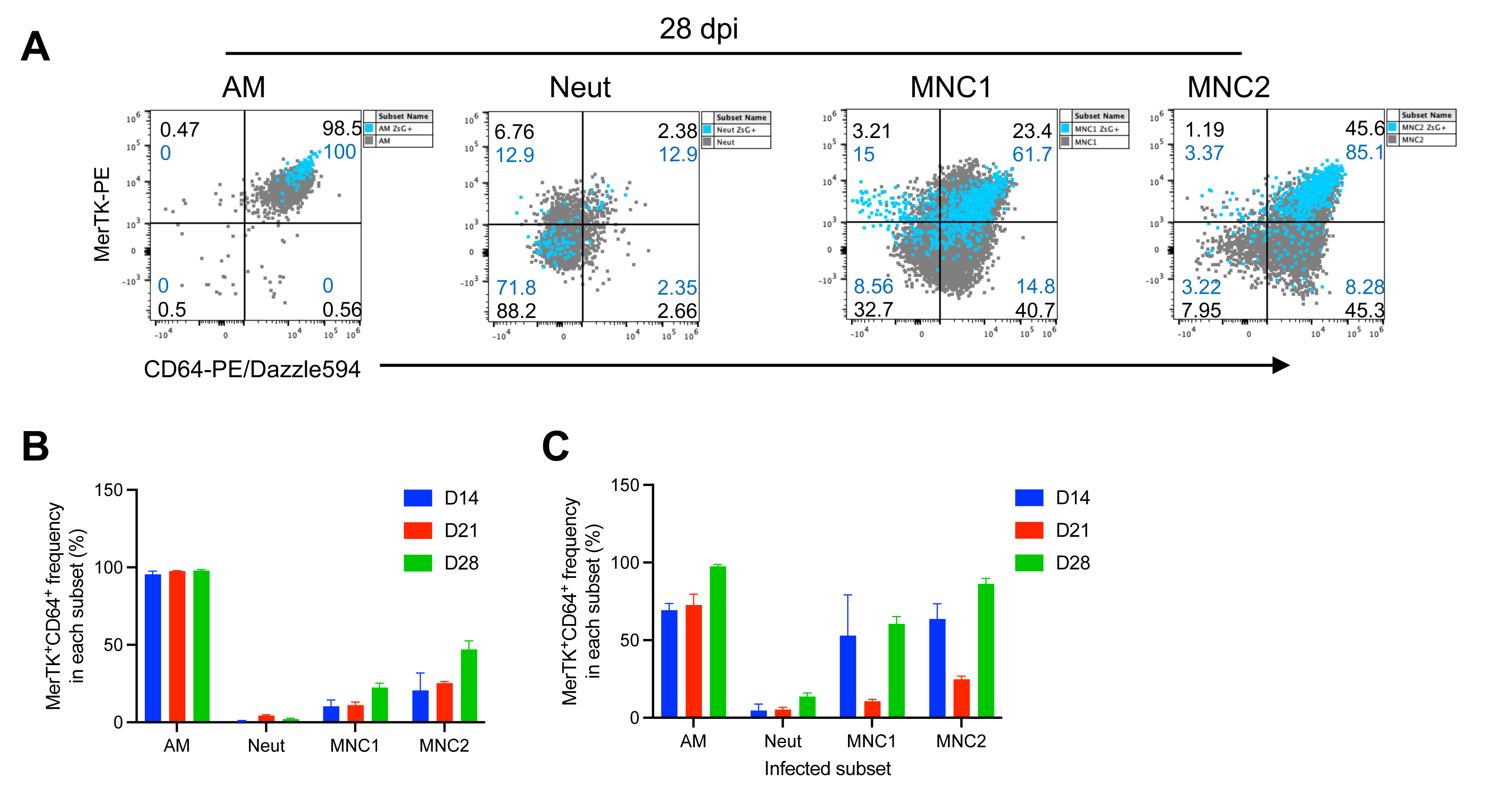

Supplement: S2 Fig — (A) Representative plots of MerTK and CD64 expression on lung subsets (grey dots), or infected cells (blue dots) from each subset of mice infected with H37Rv-ZsGreen (28 dpi). Lung subsets (AM, Neut, MNC1, and MNC2) were defined using gating strategy shown in Figure S1A, then were further analyzed for expression of MerTK and CD64. (B) MerTK+CD64+ frequency in each subset from mice infected with H37Rv-ZsGreen for 14–28 days of infection. (C) MerTK+CD64+ frequency of infected cells in each infected subset from mice infected with H37Rv-ZsGreen for 14–28 days of infection. Results are presented as mean ± SD of 4–5 mice. (TIF) [file ppat.1012205.s002.tif]

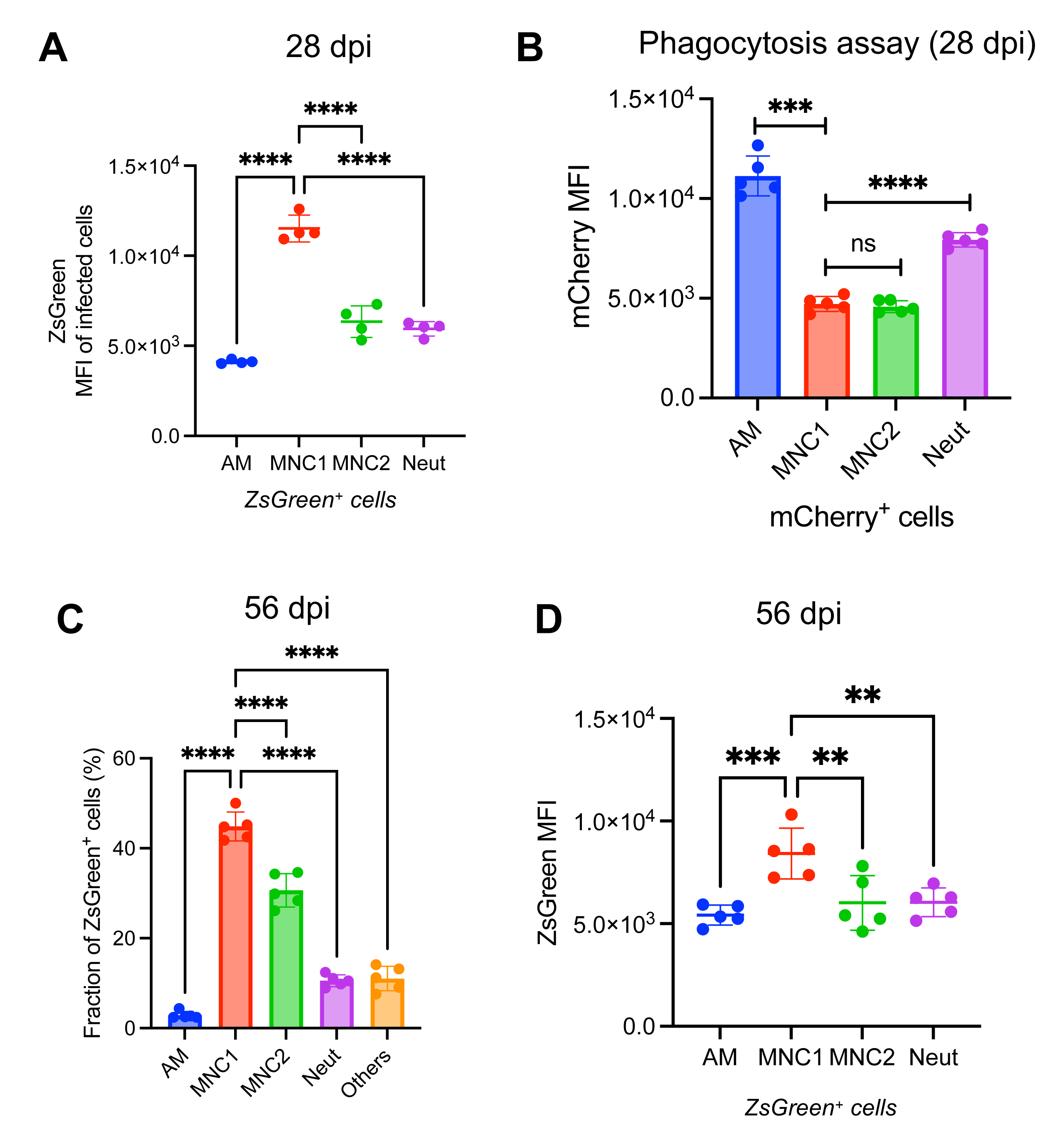

Supplement: S3 Fig — C57BL/6 mice were infected with low-dose aerosolized Mtb. At 28 dpi or 56 dpi, mouse lungs were harvested for flow cytometry analysis. (A) ZsGreen MFI of infected subsets from mice infected with H37Rv-ZsGreen (28 dpi). (B) MNC1 and MNC2 have a similar Mtb phagocytosis capacity, but lower than AM and neutrophils ex vivo. See Method detail. (C) Flow cytometry was used to analyze the subset population distribution of infected cells in mouse lungs infected with H37Rv-ZsGreen (56 dpi). (D) ZsGreen MFI of infected subsets from mice infected with H37Rv-ZsGreen (56 dpi). Results are presented as mean ± SD of 4–5 mice. *p<0.05, **p<0.01, ****p<0.0001 by one-way ANOVA. (TIF) [file ppat.1012205.s003.tif]

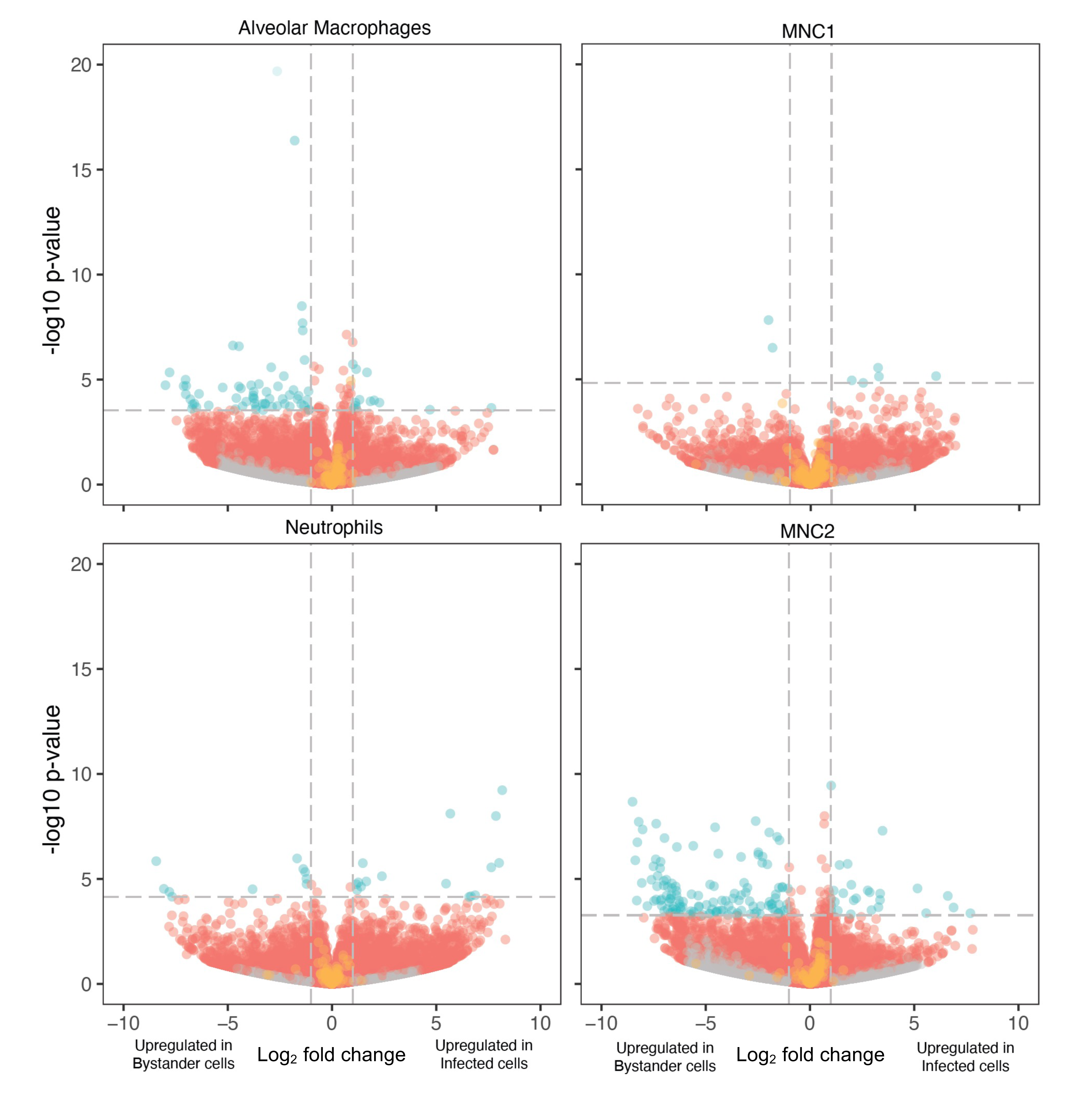

Supplement: S4 Fig — The green dot indicates significant genes with an adjusted p-value ≤ 0.05 and a |log2 fold change| ≥ 1, the red dot indicates non-significant genes, and the grey dot indicates genes filtered out of the analysis based on the Crooks index. The number of differentially expressed genes is 100, 7, 184 and 29 for Mtb-infected vs bystander AM, MNC1, MNC2 and Neutrophils, respectively. (TIF) [file ppat.1012205.s004.tif]

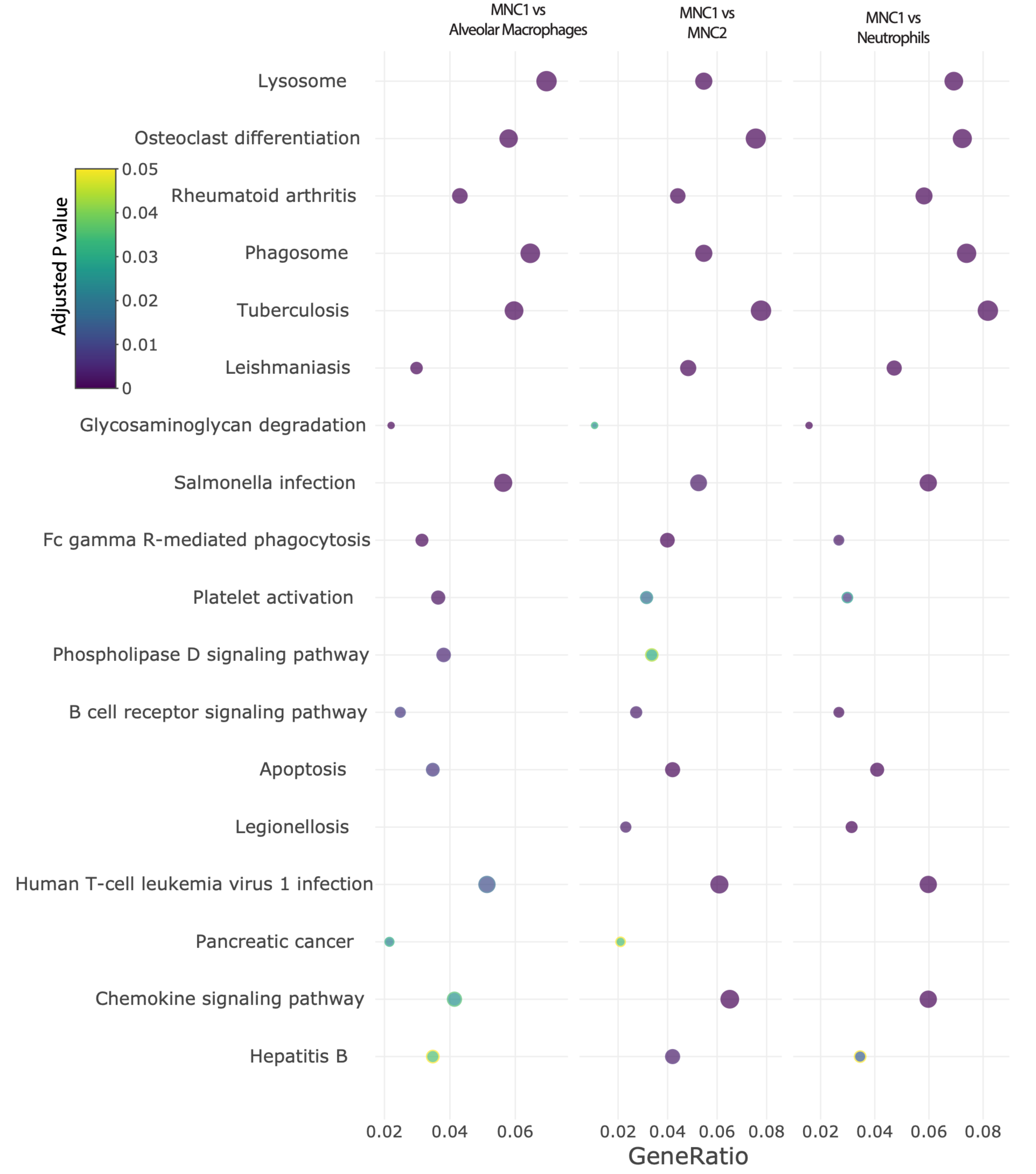

Supplement: S5 Fig — The color represents the adjusted p values, the graph is ordered by descending values for MNC1 vs AM, while the dot size is proportional to the gene count. (TIF) [file ppat.1012205.s005.tif]

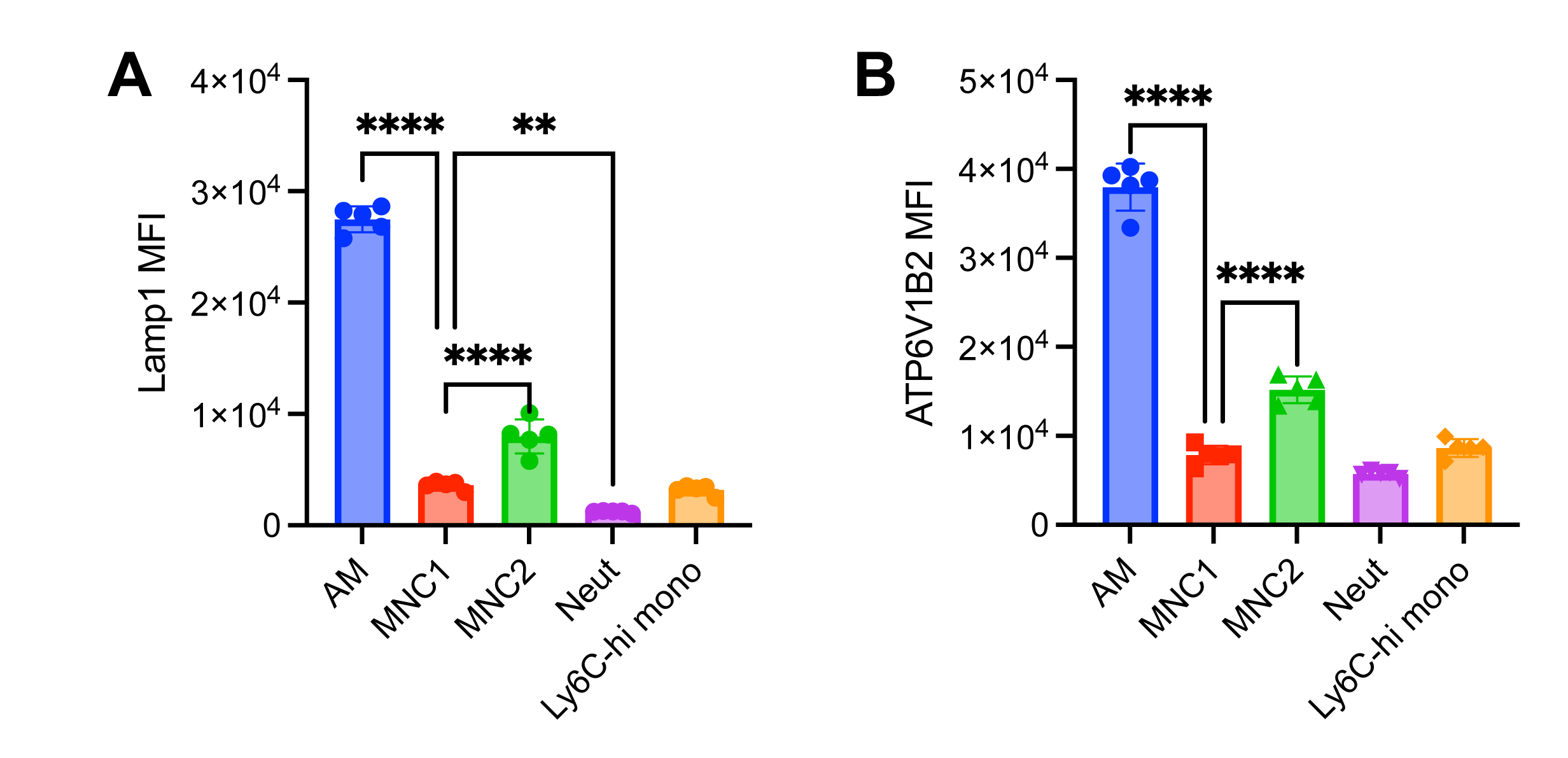

Supplement: S6 Fig — C57BL/6 mice were infected with low-dose aerosolized Mtb H37Rv-ZsGreen. Mouse lungs were harvested for flow cytometry analysis at 56 dpi. (A) LAMP1 MFI of lung subsets from H37Rv-ZsGreen-infected mice (56 dpi). (B) ATP6V1B2 MFI of lung subsets from H37Rv-ZsGreen-infected mice (56 dpi). Results are presented as mean ± SD of 4–5 mice. **p<0.01, ****p<0.0001 by one-way ANOVA (“not significant” was not shown). (TIF) [file ppat.1012205.s006.tif]

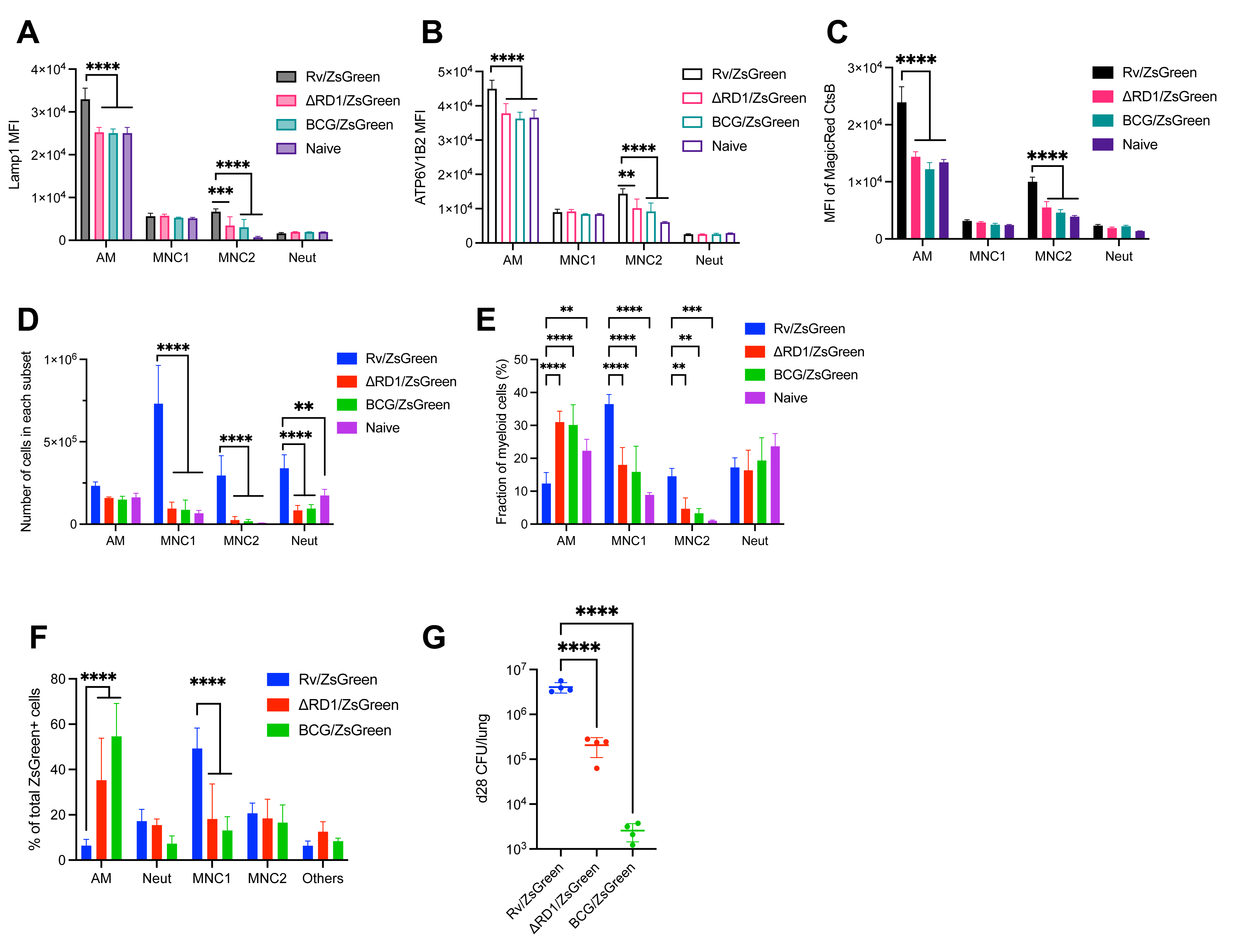

Supplement: S7 Fig — C57BL/6 mice were infected with one of the three ZsGreen expressing strains via aerosol infection: Mtb H37Rv, Mtb H37Rv:△RD1, or M. bovis BCG. At 28 dpi, lungs were harvested for flow cytometry analysis or CFU assays. Naïve mice were uninfected. (A) LAMP1 MFI of lung subsets from naïve mice and infected mice (28dpi). (B) ATP6V1B2 MFI of lung subsets from naïve mice and infected mice (28dpi). (C) MFI of fluorogenic CTSB product for lung subsets from naïve mice and infected mice (28dpi). (D) Number of cells per subset from naïve mice, and mice infected with the indicated mycobacterial strains (28 dpi). (E) Subset fractions of total myeloid cells (28 dpi). (F) Frequency of cell types in total infected cells (28 dpi). (G) Lung CFU for mice infected with different mycobacterial strains (28 dpi). Results are presented as mean ± SD of 4–5 mice, representative of 2 independent experiments. **p<0.01 ****p<0.0001 by two-way ANOVA (A-F), or one-way ANOVA for (G). (“not significant” was not shown). (TIF) [file ppat.1012205.s007.tif]

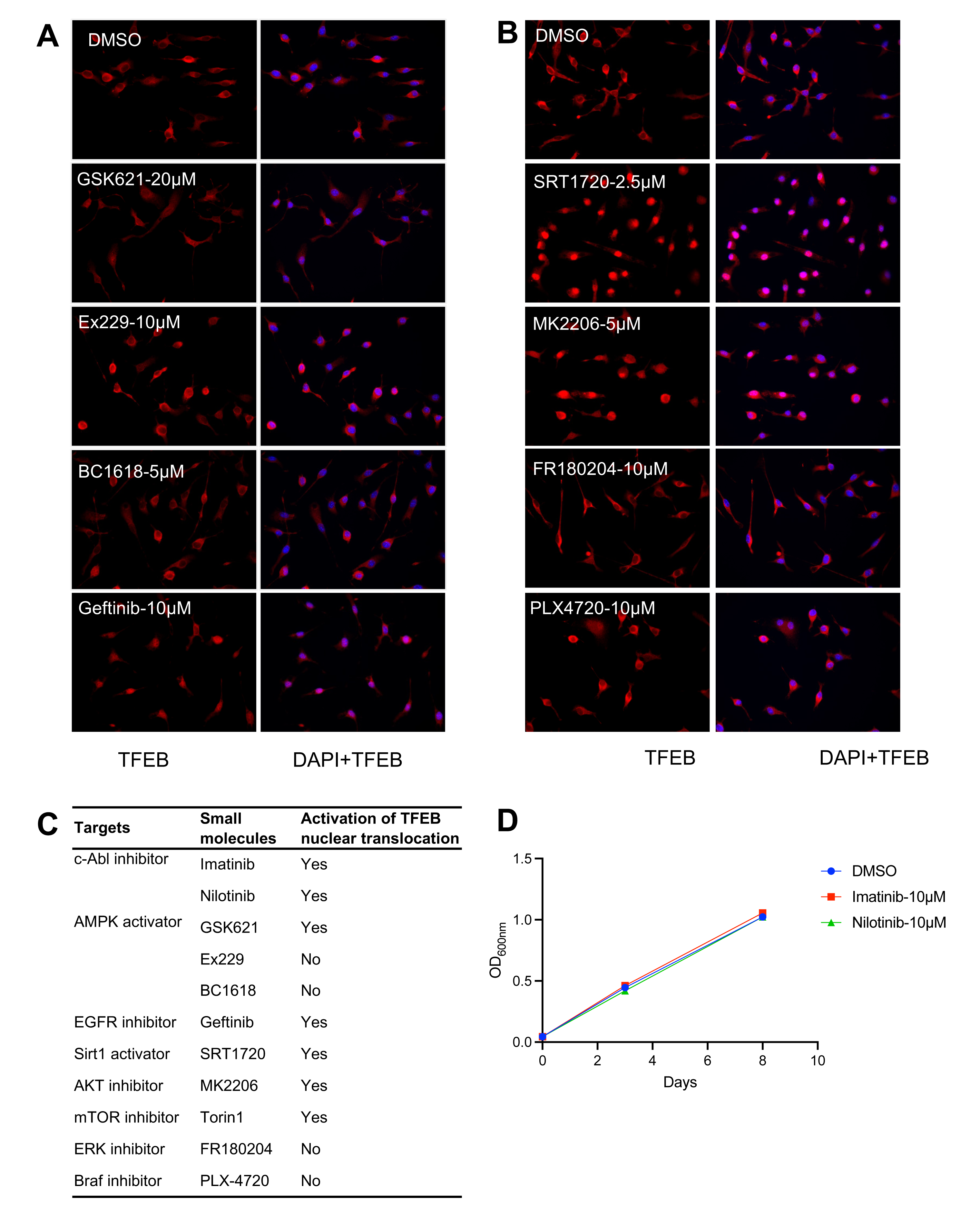

Supplement: S8 Fig — (A-B) BMDM were treated with indicated small molecules for 4h, then stained with DAPI and anti-TFEB for fluorescent microscopy. (C) Summary of the effect of small molecules on the activation of TFEB nuclear translocation. (D) Imatinib or Nilotinib do not inhibit H37Rv growth in 7H9 media. Three replicates per condition. Results are presented as mean ± SD, representative of 2 independent experiments. (TIF) [file ppat.1012205.s008.tif]

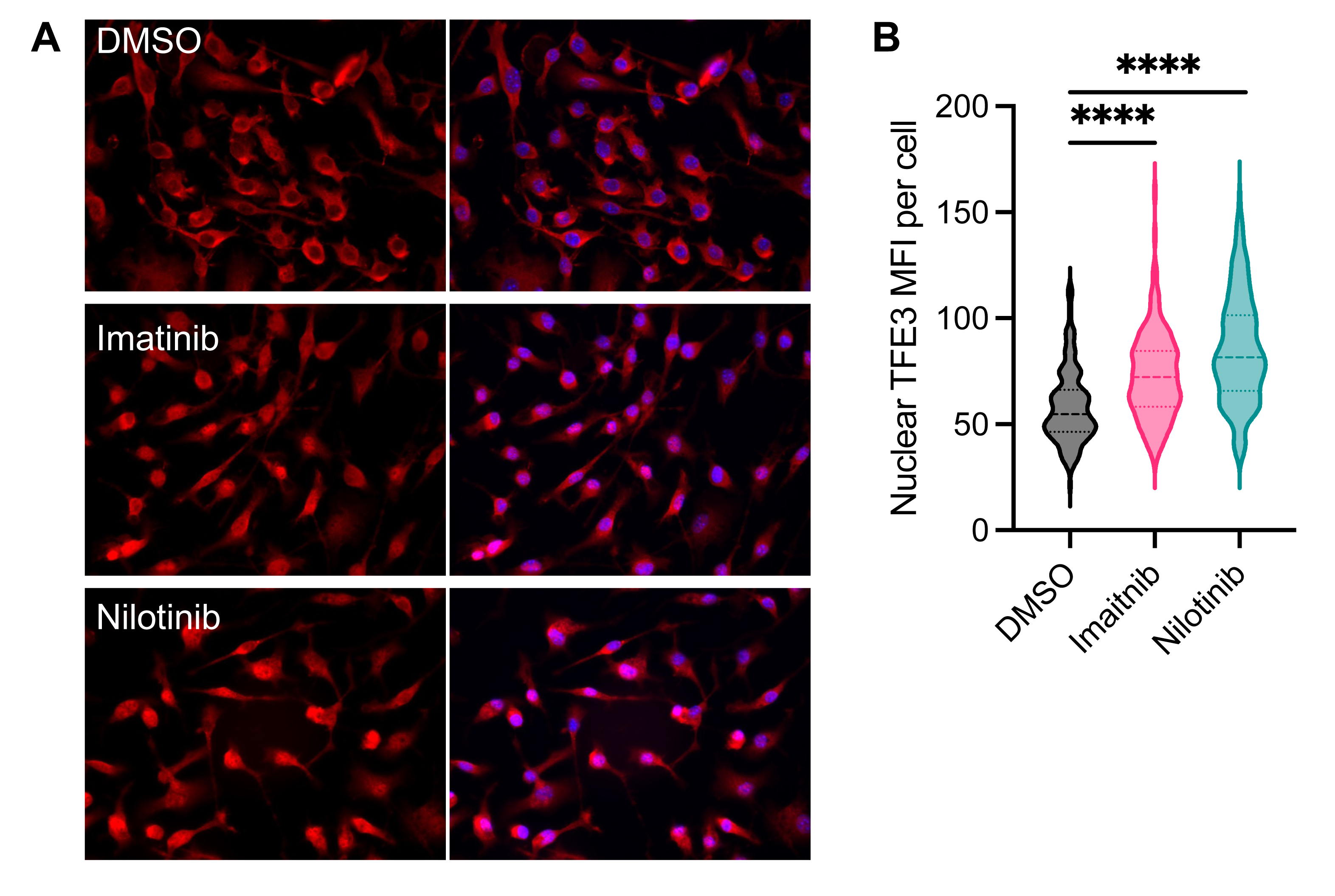

Supplement: S9 Fig — (A) BMDM were treated with indicated small molecules (Imatinib, 10 μM; Nilotinib, 10 μM) for 24h, then stained with DAPI and anti-TFE3 for fluorescent microscopy. (B) Quantification of nuclear TFE3 MFI per cell from >127 cells for each condition in (A) using ImageJ. (TIF) [file ppat.1012205.s009.tif]

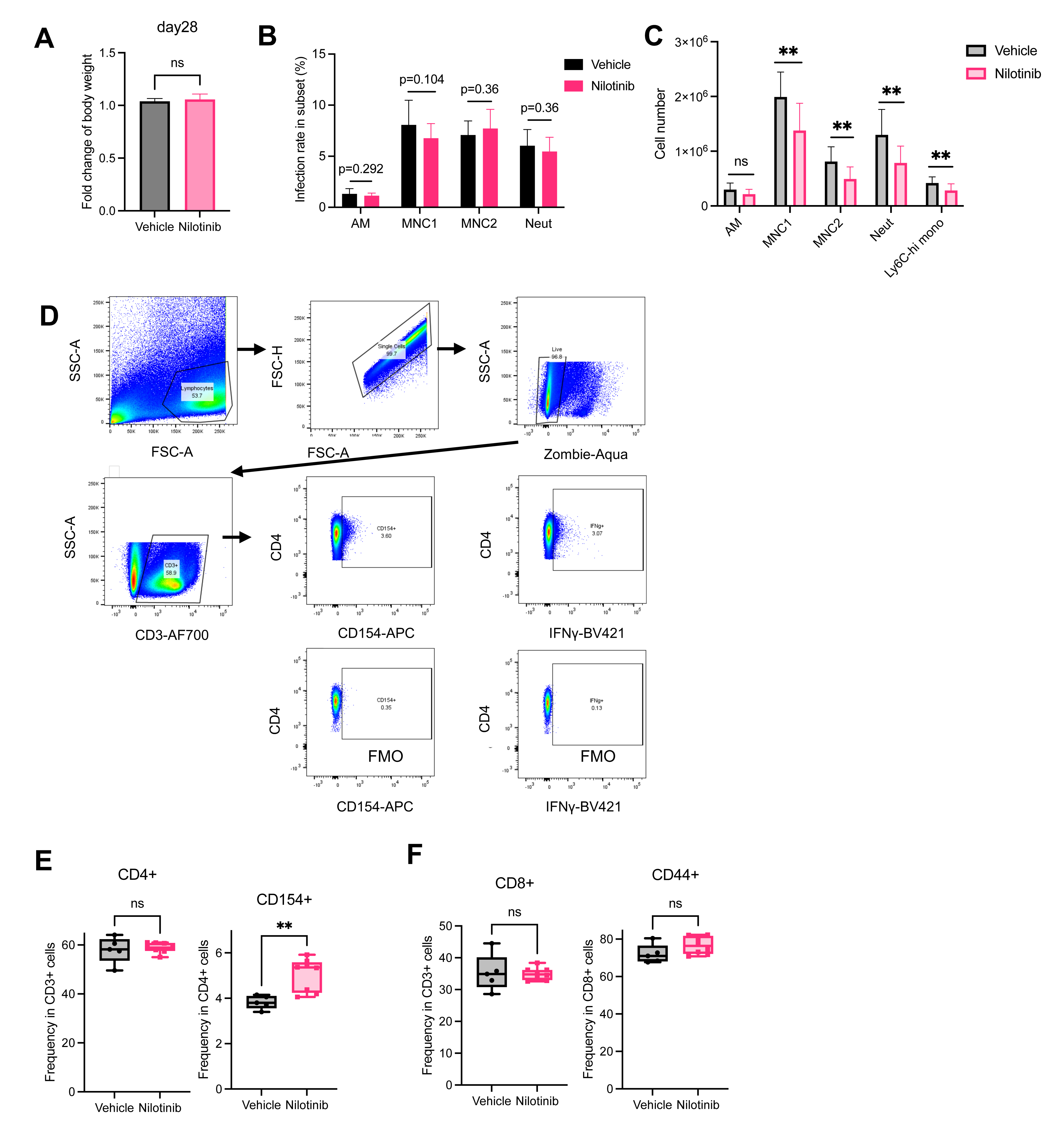

Supplement: S10 Fig — Mice were infected with low-dose Mtb H37Rv-ZsGreen via aerosol, followed by treatment with vehicle or nilotinib (20mg/kg/day) intraperitoneally, 5 days/week, a total of 15 doses. Treatment was beginning on 7 dpi and ending on 27 dpi. Lungs were harvested for different assays at 28 dpi. (A) Fold change of mouse body weight on 28 dpi relative to that on 7 dpi. (B) Frequency of Mtb infected (ZsGreen+) cells in each subset (28 dpi). (C) Total number of cells in each lung subset from infected mice treated with vehicle or nilotinib (28 dpi). (D) Gating strategy for defining lung CD4+ T cells (28 dpi). (E) Frequency of lung CD4+ T cells, and frequency of CD154+ cells in lung CD4+ T cells (28 dpi). (F) Frequency of lung CD8+ T cells, and frequency of CD44+ cells in lung CD8+ T cells (28 dpi). Results are presented as mean ± SD of 5–8 mice, representative of 2 independent experiments. *p<0.05, **p<0.01 by unpaired Student’s t-test. ns: not significant. (TIF) [file ppat.1012205.s010.tif]
