# Supplementary material for: Mycobacterium tuberculosis resides in lysosome-poor monocyte-derived lung cells during chronic infection
Source: PLoS Pathog. 2024 May 3;20(5):e1012205. doi: 10.1371/journal.ppat.1012205 (PMC11095722; doi:10.1371/journal.ppat.1012205)
Supplement: Attachment — Submitted filename: Letter with point by point responses.docx [file ppat.1012205.s014.docx]

11 April, 2024

Helena Boshoff

Section Editor

PLOS Pathogens

Michael Malim

Editor-in-Chief

PLOS Pathogens

Dear Drs. Boshoff and Malim:

Thank you for the opportunity to submit an extensively revised version of our manuscript, PPATHOGENS-D-24-00044. We appreciate the thorough critiques by the reviewers, and have revised the manuscript accordingly, including with additional results.

Following are our point by point responses to each of the comments by the three reviewers. We anticipate that the additions and edits we have made, our manuscript will now be acceptable for publication.

Sincerely,

Joel D. Ernst, M.D.

Professor of Medicine

Chief, Division of Experimental Medicine

on behalf of the authors

**Editor's comment:**

It seems important to study some of the lysosome biology in careful detail including analysis of the MNC1 (vs MNC2) lysosomes with a protein expression-independent analysis. Including a comparison of MNC2 lysosomes as suggested by reviewer 2 in some of the key figures could address the differences in bacterial load in the different cell subsets. Answering the question as to the ability of AM to control replication of MTb would be an important contribution to the field. The finding that RNAseq analyses could not distinguish between the infection status of the cells seems important to evaluate. Finally, the question as to non-lysosomal effects of the inhibitors can likely be addressed.

**Response:**

As detailed below, we have added results from an expression-independent analysis of lysosome abundance, and have added results for MNC2 in the key figures and text. We have also added a paragraph discussing the ability of AM to control Mtb replication, placing our new findings in the context of previously-published findings. Likewise, we have provided comparison of our data on transcriptional differences between infected and bystander cells from the lungs with those of other studies and added information on the differentially expressed genes in each lung cell subset, and we have qualified our results in the context of potential non-lysosomal effects of kinase inhibitors.

**Responses to Questions**

**Part I - Summary**

Please use this section to discuss strengths/weaknesses of study, novelty/significance, general execution and scholarship.

**Reviewer #1**: Zheng et al., describe a population of monocyte-derived cells that form an important reservoir of Mtb infected cells during chronic infection. These monocyte-derived cells carry higher levels of bacteria and represent a highly permissive niche for Mtb replication, driven through the limited lysosome number observed in these cells. The authors further show that reduced lysosomal trafficking is due to lower levels of TFEB in this cell population and that pharmacologically enhancing lysosome levels through treatment with c-Abl inhibitors can reduce bacterial numbers. The experiments in the manuscript are very well done and nicely illustrate their point. The manuscript itself is engagingly written and reasoned. The findings will be highly interesting to both the tuberculosis community and the wider pathogenesis community as well as researchers working on lysosomal biogenesis in both infectious and non-infectious contexts. Overall, this is a quite strong manuscript with only small weaknesses to be addressed.

**Reviewer #2**: This study by Zheng et al. highlights a profound difference between two macrophage/monocyte populations in the lung, contrasting the resident alveolar macrophage and a particular recruited monocyte population (MNC1) with regards to their lysosome biology. Specifically, they find that, at later timepoints (Day 28), MNC1 harbor more viable bacteria than other myeloid subsets and that this can at least partially be attributed to their reduced lysosome levels and lysosomal function. They can boost lysosome biogenesis pharmacologically and show that this has significant, albeit modest, effects on reducing bacterial burdens across cell types. The studies are well executed and thorough with their investigation of this particular pathway.

**Reviewer #3**: This is an interesting study that look at lysosomal activity of monocytes recruited in the lungs of infected mice in the context of tuberculosis. There is a lot of novelty and interest in these findings as there has been very little in the literature regarding lysosomal activity in immune cell subsets in vitro and in vivo. The use of functional experiments to show lysosomal activity is highly appreciated, given that most studies will only show gene expression data. There are however several interpretations of the work and approaches that are not really supported by the data and in some cases incorrect.

**Part II – Major Issues: Key Experiments Required for Acceptance**

Please use this section to detail the key new experiments or modifications of existing experiments that should be absolutely required to validate study conclusions.

Generally, there should be no more than 3 such required experiments or major modifications for a "Major Revision" recommendation. If more than 3 experiments are necessary to validate the study conclusions, then you are encouraged to recommend "Reject".

**Reviewer #1:** A central intellectual thrust is the decrease in lysosome number in the MNC1 population. While the authors demonstrate reduced LAMP1 staining, as they note, this could be a product of either decreased lysosome number or decreased expression/intensity of LAMP1 per lysosome. It would further buttress their argument to use an expression-independent approach to enumerate lysosome number (e.g. feeding fluorescent dextran to the MNC1 population).

**Response**: In response to the suggestion, we have added data from an experiment in which cells from lungs of infected mice endocytosed fluorescent dextran and were then incubated for an additional hour to allow the dextran to traffic to lysosomes. As shown in revised Figure 5F, compared with MNC1, the MFI of dextran fluorescence in alveolar macrophages cells was 12-fold higher, while the MFI of dextran fluorescence in MNC2 cells was intermediate between that of alveolar macrophages and that of MNC1 cells. We are cautious in our interpretation of these data, since differences in the rate of endocytosis could yield similar results. However, the results are consistent with those of our other assays of lysosome quantity and content, and the combined data strongly support the conclusion that MNC1 cells are deficient in lysosomes when compared with the other cell subsets in the lungs of *M. tuberculosis*-infected mice (Page 9, line 285-291).

**Reviewer #1:** While less critical, the authors might also consider performing additional staining with fluorescent dyes/sensors to get an approximate pH of the lysosomes within the MNC1 fraction compared to other fractions.

**Response**: We appreciate this comment and agree that it would add strength to our conclusions. However, the most valid approaches to determining intralysosomal pH depend on use of ratiometric analysis using pH-sensitive fluorescent dyes in live cells. Unfortunately, we do not have the required fluorescence microscopy capability in our BSL3 facility, so we have not been able to perform these studies. We do show evidence using pH-sensitive cresyl violet (Figure 4, Panels D, E, and F) that lysosomes of alveolar macrophages are more acidic than lysosomes of MNC1 cells.

**Reviewer #2:** One major comment throughout the manuscript is that the authors heavily focus all comparisons on MNC1 vs AM. The data are abundantly clear and convincing that MNC1 show a multitude of lysosome defects as compared to AM, and yet the authors are also claiming that MNC1 are more permissive to Mtb than MNC2 without showing strong differences in lysosome biology between these two subsets. While this may be a difficult bar to achieve given that the two populations are clearly closely related, it begs the question as to why the viability of Mtb shown in Fig 1E still seems to be strongly different between the two monocyte populations, with only modest differences in lysosome biology shown throughout the paper (mostly in supplemental figures, ie Fig S6 and S7). It would be helpful if MNC2 were included in many of the early lysosome defect figures, such as Fig 3 and 4, alongside MNC1 and AM data. Perhaps the differences are small but persistent enough to explain how Mtb could still be controlled in the MNC2 subset. If the differences are not apparent, the authors should comment on other reasons there might be Mtb viability differences between MNC1 and MNC2.

**Response:** We appreciate the suggestion to include data on MNC2 cells, which are intermediate between AM and MNC1 with regard to their ability to promote survival or killing of Mtb (Fig 1); and their lysosome content and quality. We have added lysosome data for MNC2 in revised Figures 3, 4, 5, and 6. The new data in the figures are also noted in the corresponding text.

**Reviewer #2:** One possible difference of interest that the authors do not comment on is the different MHCII levels between the two subsets. While CD11c expression is primarily what is used to distinguish them, MNC2 seem to be quite a bit higher for MHCII MFI than MNC1. Can the authors comment on whether this might be impacting Mtb control in MNC2 on top of lysosome deficiencies? Perhaps MNC1 are more permissive because they do not interact with T cells as well as MNC2? This could also contribute to why this phenotype emerges at a later timepoint that coincides with T cell responses.

**Response:** Regarding the point on differential MHC II expression, we have noted it and point out that it is consistent with results we published previously that MNC2 (then termed mDC, for monocyte-derived dendritic cells) are superior to MNC1 (then term recruited macrophages) in presenting antigen to Mtb Ag85B-specific CD4 T cells (PMID: 26764596, Fig S1). While we consider the possibility that this could contribute to the different properties of MNC1, MNC2, and AM, in this manuscript we focus on cell-intrinsic lysosome deficiency as a mechanism enabling survival of intracellular Mtb.

**Reviewer #2**: Given the precedence in the literature that AM are actually reservoirs for Mtb, it would be helpful if the authors could provide data using their live/dead reporter as to whether they corroborate or contrast these earlier findings. Are AM ineffective at killing Mtb at the earlier timepoints and there is a switch to Mtb killing at later timepoints? Or does your data suggest that they may always be more effective at eliminating bacteria than previously appreciated? This would be very useful information to clarify for the field.

**Response**: Our working model, supported by the published results of other investigators is that the propensity of alveolar macrophages to promote or restrict growth of Mtb is primarily related to the time interval following infection. We and others have reported that alveolar macrophages are highly permissive to Mtb survival and growth during the early stages of in vivo infection. Evidence of this from our own lab is published in *mBio*. 2022 13(3):e0133222; evidence from other labs is published in *Cell Host Microbe* 2018 24(3):439, *J Exp Med*. 2018 215(4):1135, *Sci Immunol*. 2019 4(37):eaaw6693, and *J Exp Med*. 2021 218(9):e20210615. In contrast, following development of adaptive immune responses and recruitment of monocyte-derived cells to the lungs in response to *M. tuberculosis* infection (>~21 days post infection), alveolar macrophages represent a small fraction of the lung cells harboring mycobacteria: *J Immunol*. 2007 Aug 15;179(4):2509, *PLoS Pathog*. 2020 Jun 16;16(6):e1008621. This is partially attributable to the large influx of monocyte-derived cells (*J Immunol*. 2008 Nov 1;181(9):6349, *J Immunol*. 2007 Aug 15;179(4):2509, *PLoS Pathog*. 2018 Oct 26;14(10):e1007154, and *PLoS Pathog*. 2020 Jun 16;16(6):e1008621).

We should emphasize that a major contribution in our present manuscript is the new evidence that at a time point after full development of adaptive immune responses, alveolar macrophages exhibit the ability to restrict and kill virulent *M. tuberculosis*, which likely contributes to the observation that they represent a minor fraction of the cells harboring mycobacteria in the lungs at this and later time points post infection.

Since submission of our original manuscript, another publication reports strong evidence that alveolar macrophages are highly plastic and exhibit phenotypically distinct characteristics during the course of *M. tuberculosis* in vivo: *PLoS Pathog* 2024 Jan 18;20(1):e1011871.

We have added text to the Discussion that reflects the points above, to clarify that the ability of alveolar macrophages to permit or restrict *M. tuberculosis* evolves over the time of infection, and that they are permissive early in infection and become restrictive at later time points (Page 14, lines 457-465).

**Reviewer #2**: It was surprising that the lung myeloid cells did not segregate by infection status in the RNAseq analysis given the published literature that infected and bystander cells do strongly segregate at earlier timepoints (Rothchild et al, up to 10 days post-infection, Pisu et al, 21 days post-infection). Do you think this is a timepoint-specific phenomenon where the transcriptional changes are shut off during chronic infection? Or that bystander cells experience so much antigen stimulation that they fully mimic infected cells at these later timepoints? It would be helpful to show whether you can replicate the published findings of clear transcriptional segregation between bystander and infected cells at earlier timepoints to ensure there isn’t a technical difference in the analyses and to highlight the unique nature of these chronic timepoints.

**Response**: We appreciate the reviewer’s attention to this issue. We do find differentially expressed genes according to the presence or absence of intracellular Mtb in each cell subset, but the differences are small compared with the differences that distinguish the cell subsets from one another. We now include Volcano plots that show differentially expressed genes (DEGs) between infected cells and bystander cells for each lung cell subset (Figure S4). The number of DEGs is 100, 7, 184 and 29 for infected vs bystander AM, MNC1, MNC2 and neutrophils, respectively (updated in the legend of Figure S4). The number of DEGs identified in this study is similar to those reported in published studies by other investigators. The study by Rothchild et al (PMID: 31350281) identified 196-252 DEGs in the comparison between infected AM with AMs from uninfected mice (the comparison was not of infected vs bystander cells from infected mice) at various timepoints up to 10 days post-infection. The work by Pisu et al (PMID: 34292313, Table S31 and S33) identified 54 DEGs for the subset they designate IM2, and 41 DEGs for IM3. Furthermore, our results of subset clustering analysis are consistent with published work where they also showed that lung myeloid cell subsets do not segregate by infection status 21-25 days post infection (see Figure1e in PMID: 34292313, Figure 2B in PMID: 38029747).

**Reviewer #2**: In the final figure, it is important to determine whether these inhibitors are ultimately affecting Mtb viability in MNC1 using the same live/dead reporter analysis shown in Figure 1. In addition, instead of plotting # infected cells/subset as in Fig 7C, can the authors plot this as % infected of each subset? That would help visualize how well the drug is impacting bacterial load in each type of immune cell. Finally, given the modest effects of nilotinib, can the authors comment on why this drug was chosen over imatinib? Maybe that drug would have performed better.

**Response**: Although MNC1 exhibit the lowest levels of lysosome abundance, content, and activity of the 3 mononuclear cell subsets in the lungs of Mtb-infected mice, we did not anticipate that an effect of nilotinib on lysosome biogenesis and handling of Mtb would be limited to MNC1. Indeed, as shown in Fig 8C (formerly 7C), the number of infected cells was reduced in all 3 cell subsets in mice treated with nilotinib, consistent with the overall reduction of Mtb cfu shown in Fig 8B. We also note that nilotinib treatment had the additional effect of reducing the number of inflammatory cells (MNC1, MNC2, monocytes, and neutrophils) to the lungs (Fig S10C). We now include a figure to show the frequency of infected cells in each subset (Figure S10B), which reveals a slight but not significant reduction of infected frequency for MNC1 subset in the nilotinib group (see Page12 line 385-387).

While in vitro data do not always reflect in vivo effects, our in vitro data in Figure 7K-7L show that nilotinib activated lysosome function and restricted Mtb replication in BMDM, which is consistent with the increase of lysosome function in MNC1 and MNC2.

Figure 7E-7G reveal that Nilotinib is more effective than imatinib in activating lysosome activity of macrophages, and Figure 7K shows that nilotinib is slightly better than imatinib in controlling Mtb intracellular growth in cultured BMDM. Therefore, we chose nilotinib for the in vivo study (see Page11 line 381-382).

Regarding the suggestion that we repeat studies using the live/dead reporter plasmid in cells isolated from nilotinib-treated mice, we regret that we are unable to perform those studies in the foreseeable future. Our lab recently located to a newly-constructed research building and a new BSL3/ABSL3 facility, with a new cell sorter whose operation is distinct from the sorter used to generate the data in Figure 1. Considering the effort that was required to optimize conditions for live sorting and downstream analyses with the previously-available sorter, we anticipate that it will be at least 6 months before we are able to perform the suggested experiments with the rigor required.

**Reviewer #3**: The use of a live dead reporter is very interesting. The system is based on doxycycline, a drug that eventually cannot reach all tissue environments. So, I would be more careful and consider the double positives active bacteria.

**Response**: We appreciate this comment and have added this point as a potential limitation (page 5, line 168-170). Because we realized the potential shortcomings of the doxycycline live/dead assay, we additionally performed the studies whose results are shown in Figure 1G, in which we live-sorted EGFP^+^ cells from lungs of mice infected with *M. tuberculosis* expressing EGFP and plated the sorted EGFP^+^ cells to quantitate live bacteria as colony-forming units (cfu) per 1,000 EGFP^+^ (e.g., Mtb-infected) cells (Fig 1G). The results are in full agreement with the results obtained with the live/dead assay.

**Reviewer #3:** The concept of permissive is more related to replication, so to conclude on this aspect, it could be important to re-analyse the data and look at the total intensity per cell (e.g. as shown in Fig 1E).

**Response**: We are cautious in our interpretation of the results shown in Figure 1 because we are not aware of technology currently available to determine whether all of the live bacteria in a given cell are the products of bacterial replication in that individual cell. As a consequence, we don't address the topic of replication per se. If the reviewer can suggest a term that is more appropriate than 'permissive' to describe cells that harbor the highest number and highest fraction of live bacteria, we are open to using an alternative term.

**Reviewer #3:** In Figure 3G is not correct to suggest autophagy deficiency based only on this autophagy probe. The deficiency in proteolytic activity is clear and this will affect all lysosomal dependent degradation pathways. I suggest omitting the autophagy part here as it is irrelevant.

**Response**: We have removed Figure 3G as suggested.

**Reviewer #3:** Antibodies against subunits for the vATPAse are notoriously bad (for staining in flow and immunofluorescence, tissues), so I suggest the authors be extra cautious here and add some controls that the antibodies work. The other experiments in Figure 4 (e.g. cresyl violet, Cathepsin substrates etc) are enough evidence for defective lysosomal function.

**Response**: We compared isotype control antibodies and vATPase antibodies on bone marrow derived macrophages (BMDM), which confirmed that the fluorescence are more intense with anti-ATP6V1B2 and anti-ATP6V0D2 than with the isotype controls (see figure below, provided for the review only). In contrast, we did not observe positive staining with anti-ATP6V0A1 or anti-ATP6V0D1, therefore, we removed the data using these two antibodies from Figure 4. We have also added a comment “verified using isotype control” for these antibodies in the “Immunofluorescence” section. As the Reviewer notes, our other results support the conclusion that lysosome acidification, content, and activities are deficient in MNC1, and these immunofluorescence results are consistent with those other results.

Figure for reviewers only: immunofluorescence staining of BMDM using VATPase antibodies and isotype controls.

**Reviewer #3:** Nilotinib has many other effects that are lysosomal function independent. These results shown in Figures 6 and 7 are very interesting but I will be more cautions with the interpretation in the discussion.

**Response**: We acknowledge that nilotinib has many effects; some of these are due to the pleiotropic effects of inhibiting the Abl tyrosine kinase, while others are due to inhibition of other kinases. Our results indicate that nilotinib increases lysosome gene expression and lysosome activities in vitro and improves control of *M. tuberculosis* in vivo. We have added a comment to qualify our conclusions in the Discussion (page 13-14, lines 453-455).

**Part III – Minor Issues: Editorial and Data Presentation Modifications**

Please use this section for editorial suggestions as well as relatively minor modifications of existing data that would enhance clarity.

**Reviewer #1**: Line 75-78 - It’d be helpful to break up this sentence/streamline to make more clear

**Response**: We have modified this as suggested (Page 3 line 75-78).

**Reviewer #3**: Line 324-329, line 414-416 – The lower recruitment of MNC1 and other cell types is considered as being evidence of RD1-dependent recruitment, however, given the lower burden in RD1 and BCG infected animals, these effects could also be secondary to the lower burden/decreased RD1-dependent growth. This should be discussed as an alternative possibility, as in the absence of matched infection burdens, it is hard to definitively establish that recruitment effects are directly dependent on RD1.

**Response:** We have added a comment acknowledging that the differences in cell recruitment could be attributable to the differences in bacterial burdens in mice infected with wild-type vs ESX-1-deficient Mtb (Page10, line 338-339).

**Reviewer #3**: Figure 4 and S10 – Reorder the panels if possible.

**Response**: Figure 4 and S10 were reorganized as suggested.

**Reviewer #2**: It is appreciated that the authors have attempted to place their gating strategy into context with prior manuscripts and acknowledge heterogeneity in the MNC2 population, especially given the RNA-seq results that show the presence of DC as well as monocyte genes. Fig S2 is somewhat confusing as it attempts to compare the MNC1 and MNC2 as defined in this manuscript with prior studies using Mer-TK and CD64, but it is unclear how the cells in Fig S2 are pre-gated (if they are pre-gated?) and how this gating was subsequently applied to Fig S1 (as is mentioned in the figure legend). The figure shows that many cells fall outside the Mer-TK and CD64 gate, but among Mtb-infected cells it does seem to define the cells quite well, except for some CD64-negative cells in the MNC1 population. As such it would be nice to see additional flow cytometry using known markers to distinguish conventional DC from monocytes, including CD26, CD64, and MAR1, as in Bosteels et al, Immunity 2020. CD88 can also been used to distinguish monocyte-derived cells from DC.

**Response**: To clarify this point, we have updated the figure legend for Figure S2, read as “Lung subsets (AM, Neut, MNC1, and MNC2) were defined using gating strategy shown in Figure S1A, then were further gated using MerTK and CD64.”

Due the limited parameter capacity of the sorter in our BSL3 facility, we were not able to accommodate more markers in our panel for sorting. Moreover, other studies have indicated that classical DC are much less abundant than monocyte-derived cells in the lungs of Mtb-infected mice (PMID: 34292313). We continue to seek informative markers that discriminate these cell subsets and note this point (page 6, line 201-202).

**Reviewer #2**: What was the gating based on for MNC1 vs 2 for CD11c? Seems like the natural break in the populations is substantially lower.

**Response**: We have examined multiple other markers and so far we have been unsuccessful in identifying markers that distinguish the two subsets with higher resolution. Ongoing studies are directed at understanding the developmental relationship of the two subsets.

**Reviewer #2**: Include negative control for representative flow cytometry plots in Fig S1 to determine how much fluorescent background might be present in each bacterial strain, especially given that the zsStrain seems to induce much higher levels of infection than either the mCherry or GFP strains.

**Response**: Negative controls were added in Fig S1 as suggested.

**Reviewer #2**: Clarify if Fig 3B is showing MFI background-subtracted for the “no substrate” histograms to show change in fluorescence upon addition of substrate. While the difference between cell types is clear, they do have slightly different fluorescence signals at baseline that should be accounted for. Same comment for the autophagy figure (Figure 3H). In the latter, there appears to be much stronger induction of autophagy than cathepsin activity, but it is still lower in MNC1 than AM.

**Response**: Reviewer 2 is correct, MFI in Fig3B is background-subtracted using the FMO control. We have updated this in the “Quantification and statistical analysis” section, read as “MFI data shown for lung subsets were net MFI after subtracting the background MFI of the corresponding FMO control.”

**Reviewer #2**: It would be nice to see representative flow plots for the staining in Fig 4C.

**Response**: Representative flow plot for ATP6V1B2 is now shown in Figure 4B.

**Reviewer #2**: Page 8, line 248. There is a typo, with “different” needing to be deleted.

**Response**: Thanks for the comment. We deleted “different”.

**Reviewer #2**: Did the authors use intravenous antibody labeling prior to euthanasia to differentiate monocytes in the parenchyma versus vasculature? There could be differences in such populations and it would be interesting if any of these lysosomal deficiencies were unique to parenchyma-resident monocytes.

**Response**: Due to limited parameters that could be ascertained simultaneously by the BSL3-contained cell sorters we used for these studies, we were unable to use i.v. anti-CD45 to distinguish parenchymal from intravascular cells. However, our previous characterization of the monocyte-derived cells in the lungs used i.v. anti-CD45 and analytical flow cytometers with more detectors and revealed that the large majority of MNC1 and MNC2 cells (in that publication, termed ‘recruited macrophages’ and ‘monocyte-derived dendritic cells’ respectively) are parenchymal (*PLoS Pathog*. 2018 Oct 26;14(10):e1007154).

Because of our inability to use i.v. anti-CD45 with the BSL3 cell sorters, we performed separate experiments in which we compared the perfusion protocol used in this work (PBS/EDTA via the right ventricle) to i.v. anti-CD45. This revealed that the perfusion with PBS/EDTA used in the studies here eliminates 90-95% of the i.v. anti-CD45+ cells, indicating that the results we report here predominantly involve monocyte-derived cells in the lung parenchyma.

**Reviewer #3:** In Figure 5, cytosolic will be more appropriate than extranuclear.

**Response:** We appreciate this comment. We used “cytosolic” in the legend.

**Reviewer #3**: Scalebars are missing in many images, for example 6A

**Response**: We have added the scale bars.
